# Consumers' Perception About Front of Package Food Labels (FOPL) in India: A Survey of 14 States

**DOI:** 10.3389/fpubh.2022.936802

**Published:** 2022-07-15

**Authors:** Sudip Bhattacharya, Om Prakash Bera, Vandana Shah

**Affiliations:** ^1^Department of Community and Family Medicine, All India Institute of Medical Sciences, Madurai, India; ^2^Global Health Advocacy Incubator, Mumbai, India

**Keywords:** front of package (FoP) labels, FSSAI, food labeling, nutrition, NCD and risk factors

## Abstract

**Introduction:**

Front of Package Food Labels (FoP) help consumers make healthier food choices at the point of purchase by giving details about the nutrients available in the packaged food items.

**Aim and Objective:**

A prospective multi-centric cross-sectional study was conducted in 2021 across India to evaluate the existing knowledge and attitude regarding food labels on packaged foods and beverages. Also, the objective understanding of the consumers' knowledge on different types of FoP label practiced across the world was determined.

**Methodology:**

A self-administered questionnaire was given to the respondents to gather their attitudes regarding the FoP label. Besides, they were given colored pictures of different FoP labels to seek their perception and preference for different FoP label designs.

**Results:**

Results found that packaged food and beverages were consumed by 91.3% of the participants. Awareness about the food package labeling was widely held by 95% of the participants and 88.6% of them considered this information helpful. Over half (55.4%) of the respondents considered packaged foods as healthy. Warning Labels (WL) were the most preferred food labels (93%), followed by Multiple Traffic Lights (MTL) and the difference between the two was statistically significant (*p*-value < 0.05).

**Conclusions:**

The awareness about FoP labels is low among the consumers.

**Recommendations:**

Evidence-based research is recommended regarding the knowledge and perception of people on the feasibility of FoP label design which may lay a foundation to formulate laws and policies regarding the front of pack labeling.

## Background

Non-communicable diseases (NCDs), also called lifestyle diseases, including cardiovascular diseases, cancer, chronic kidney diseases and stroke, account for 41 million deaths globally (1). While on the one hand, dietary habits are considered as significant contributors for NCDs, on the other hand, they are looked upon substantially to plan public health strategies owing to their modifiable properties (2–6). Resultantly, various public health policies have been framed and executed worldwide to help individuals change their dietary patterns (7–11). Public health authorities are working extensively to increase the awareness regarding the nutritional information through food labels provided either at front or back of the food products. Front of pack labels (FoP) help customers in making informed choices to prefer or select the food while purchasing (4–12). Furthermore, they play a vital role in stimulating the food producers to reform the food items and label the packaged food (13, 14). Evidence suggests that the introduction of FoP labeling is a cost-effective approach of achieving better health outcomes (15, 16).

Consumers must first grasp the information provided by a FoP label for it to be useful in purchasing scenarios (17). There are two types of understanding: subjective and objective comprehension. The former refers to the customer's capacity to interpret the FoP label information as intended by its creators (17), whereas the latter involves analyzing the labeling information as intended by its designers. In order to gather subjective understanding, a self-reported questionnaire is given to the consumer to study their perception regarding the comprehension of the information provided through labeling on the packaged food product. On the other side, the consumers are given a task to rank or select FoP label designs using pictures of food goods exhibiting FoP labels to gather an objective understanding of FoP label. Evidence suggests that numerous factors at the individual and manufacturer level influence practical understanding of the pack label (e.g., dietary preference, age group, education, occupation, residence, graphical design, etc.) (17). Over the last decade, various labeling designs have been created. The nutrient-specific and summary labels provide information regarding the nutrient content and food product's overall nutritional quality, respectively. Labels for individual nutrients are classified as numeric-only, color-coded and warning label groups. Reference Intakes (RIs), UK's Multiple Traffic Lights (MTL) label; and Chile's Warning labels developed in 2006, 2005, and 2016, respectively, are few examples of these three categories (18–20). Summary FoP labels are further sub-divided into scale-based graded labels and the endorsement symbols. The Australia's Health Star Rating (HSR) system and France's Nutri-Score developed in 2014 and 2017 (21, 22) are few examples. FoP labels are generally well-liked by customers and can help them become more aware of the healthiness of various foods (23–25).

Furthermore, consumers are more likely to understand interpretive labels than strictly informative ones (26). Many nations are exploring to use FoP labels as a national public health instrument. However, few researchers have found disparities in consumer knowledge and FoP label format efficacy between countries (27, 28). Besides, the review lacks the comparison **studies about different FoP labels across diverse cultural contexts**.

### Rationale

To the best of our knowledge, most of the studies conducted in the past across the globe to assess the knowledge, attitude, and practice regarding FoP labels are qualitative study, which had a very little scope to know the problem in quantitative manner. Having 2,024 samples from 14 states, i.e., the large scale survey is the first of its kind study to have been conducted in India and it adds the novelty factor to this present study.

The experimental approach was used to evaluate the existing knowledge and attitude regarding food labels on packaged foods and beverages and determine the objective understanding of the consumers knowledge on five FoP labels (HSR, MTL, Nutri-Score, RIs, and Warning symbol) practiced across the world.

## Methods

### Study Design and Setting

The current prospective multi-centric cross-sectional study was conducted between January and March 2021 in 14 states across India. The centers comprised the premier institutes of the study states.

### Sampling and Sample Size

**Figure 1:**
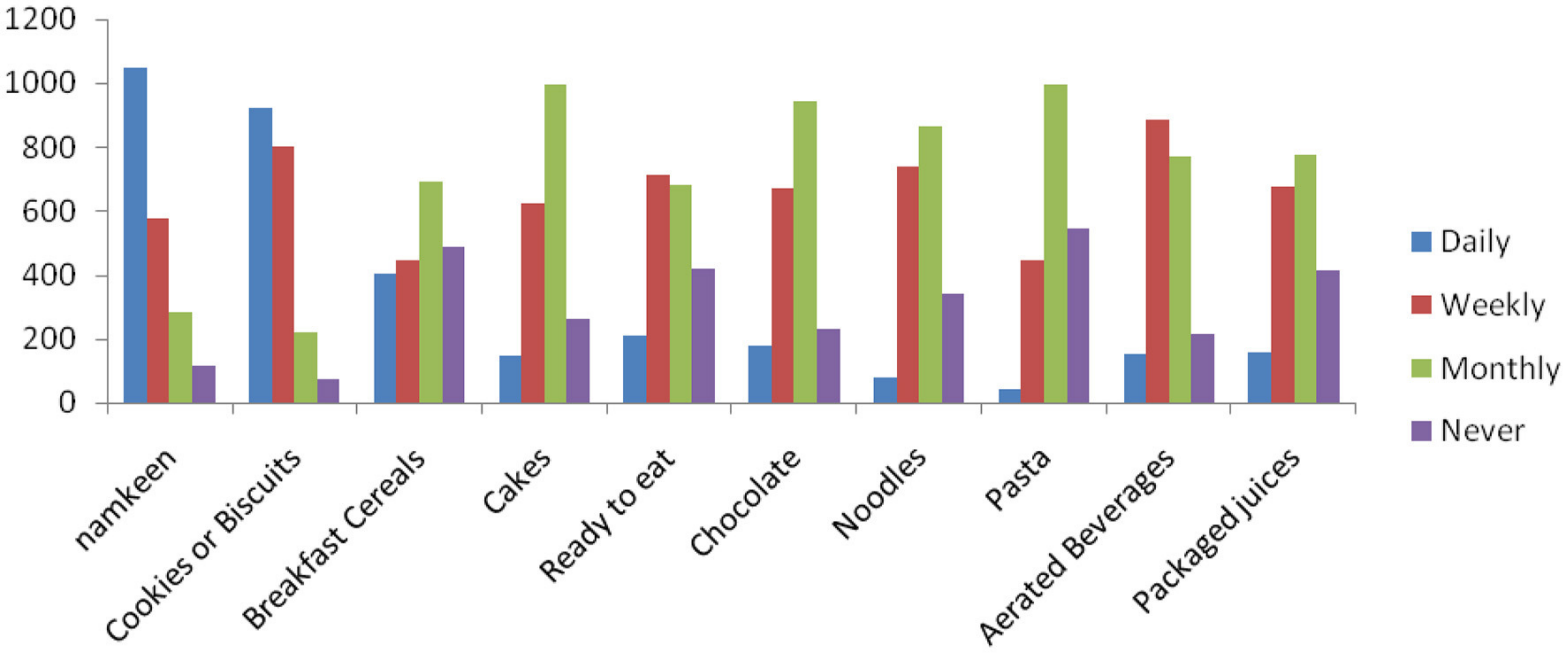
Consumption of packaged foods by the public.

Purposive sampling method was used to recruit the sample to conduct a physical questionnaire-based survey. Quota sampling design (for age), was used for the participants recruitment in each center/state. The individuals having direct relation or expertise in nutrition or any health related field were excluded from the study. In total, 2024 respondents, representing 14 states of the country, participated in the study. Figure 1 portrays the geographical representation of the participants.

### Study Tools and Data Variables

The subject experts developed a pre-tested, semi-structured questionnaire from the field of nutrition and other technical peers. It was validated before dissemination among the respondents. It was a self-administered questionnaire seeking information about the socio-demographic profile of the participants- sex, age, education, income, household composition. Further, the questionnaire assessed the respondents use of packaged foods, and type and frequency of consuming packaged foods and different FoP labels used internationally. The questionnaire also captured the acceptability of the FoP labels and the consumer's subjective and objective understanding and use of nutrition labels while choosing the food products.

### Data Collection

The duration of the survey, which data would be stored, where and for how long, who the investigators were, and the study's goal were all communicated to the participants using CHERRIES checklist. We included the participants who are literate, having knowledge of computers/mobile/internet, willing to participate and used to take packaged food in routine. We used a Google Forms-based questionnaire that has been thoroughly vetted. Each participant signed a digital consent form to participate. The lead investigator gathered data and stored it on an encrypted hard disc. Before being sent out, the electronic questionnaire (in English) was pilot tested. It was an open survey with online participant recruiting. The poll and its goals were promoted through a variety of platforms, including social media and email groups. The answers were instantly entered into a database. Our survey was completely voluntary, and no monetary rewards were offered. The questions were typed into a Google form so that the investigators could easily make sure it is correct. View rate, levels of participation, and completion percentage are examples of unique variables. We also kept logs for later analysis. To account for the non-representative sample, no approaches such as item weighting or propensity scores were applied. It took around 15–20 min for each participant to complete the offline survey. The first section comprised a few questions regarding the socio-demographic profile of the respondents. The participants were asked about the regularity of consumption of various packaged food and beverages on a 4-point scale (daily/weekly/monthly/never) and we have considered if the percentage score is below 60%, then it will be considered as low knowledge, 61–80% will be high, and >81% will be considered very high knowledge about FoP labels on food items. Then the questions seeking the attitudes of the participants regarding FoP labeling were asked such as “I like this label”; “This FoP label is useful”; “This FoP label stands out”, and “This label is easy to understand”, etc. Finally, respondents were provided colored pictures of different FoP labels and primary details were provided for MTL (Multiple traffic lights), WL (Warning Labels), HSR (Health Star Ratings), RI (Reference Intake), NS (Nutri Score) and then were asked for their perceptions of the different types FoP label they saw.

### Ethical Considerations

Ethical clearance was taken from the Institute Ethical Committee. The survey link consisted of a disclosure page explaining the purpose of the study and the intent for its subsequent publication. Survey questions could be answered after providing informed consent for participation and use of the contents of responses toward a research publication. Respondents were reassured about anonymity, confidentiality, and data security.

### Statistical Analysis

Descriptive analysis (mean, standard deviation) was done using SPSS version 21 (IBM Chicago, USA). Descriptive statistics, co-relational, group comparison, chi-square, and regression analyses will be utilized. Chi-square test of homogeneity and likelihood ratio test was applied to see the association between the consumption of the packaged foods with other socio-demographic variables.

## Results

Over three-fifths of the respondents (62%) were males, and over half of them (50.1%) were 18–29 years old. Out of the total respondents, 46% were graduates, followed by 17% of postgraduates, with more than two-thirds (70%) belonging to the urban area and over one-third (39%) representing South India. More than 60% of the respondents were employed either in govt., private or self-employed and over one-third (40%) had a monthly household income ranging from Rs. 15,000 to 50,000/- (Table 1).

**Table 1:** Demographic information of the participants.

		***n***	**%**
Gender	Female	760	37.5
	Male	1,261	62.3
	Other	3	0.1
	Total	2,024	100
Age	18–29 years	1,014	50.1
	30–50 years	874	43.2
	>50 years	136	6.7
	Total	2,024	100
Geographic zone	North India	601	29.7
	West India	298	14.7
	East India	323	16.0
	South India	802	39.6
	Total	2,024	100
Locality	Rural	617	30.5
	Urban	1,407	69.5
	Total	2,024	100
Level of education	Illiterate	40	2
	Primary school	113	5.6
	High school	295	14.6
	Intermediate	308	15.2
	Degree	931	46.0
	Post-graduate	337	16.7
	Total	2,024	100.0
Employment status	Unemployed	253	12.5
	Employed	723	35.7
	Self-employed	507	25.0
	Student	519	25.6
	Retired	22	1.1
Monthly household income	<Rs. 5,000	61	3.0
	Rs. 5,000–15,000	544	26.9
	Rs. 15,001–50,000	806	39.8
	Rs. 50,001–1,00,000	416	20.6
	>Rs. 1,00,001	69	3.4
	I don't know	128	6.3
	Total	2,024	100

Table 2 presents the knowledge and attitude of the respondents regarding the consumption of packaged foods and drinks. Packaged food and beverages were consumed by 91.3% of the respondents. We applied a chi-square test of homogeneity and found a significant difference in consuming packaged food (*p*-value < 0.05). The Likelihood Ratio Test was applied to study the difference between the consumption of packaged food and socio-demographic variables. A maximum proportion of the consumption of the packaged food was found among the participants from South and North India, and it was found to be statistically significant in terms of the graphical zone (LR = 42.915, *p*-value = 0.00001). Urban area people consumed more packaged food than rural area people (LR = 22.013, *p*-value = 0.000003). Under-graduate people consumed maximum packaged food and beverages as compared to the illiterate class (LR = 33.473, *p*-value = 0.000002). Employed people consumed maximum packaged food and beverages as compared to the retired people (LR = 74.334, *p*-value = 0.000001). Respondents having monthly income between Rs. 15,000 and 50,000/- consumed maximum packaged food and beverages. Those with income more than 100,000 or <5,000 consumed packaged food and beverages the least and the difference was found to be statistically significant also (LR = 50.730, *p*-value = 0.00001).

**Table 2:** Consumers knowledge about food labeling.

		***n***	**%**
1. Do you know food items come with food package labeling?	Yes	1,876	95.1
	No	96	4.9
2. Do you think these food package labeling serve any purpose for consumers?	Yes	1,804	91.5
	No	168	8.5
3. Where are these food package labels usually present on the food items?	Front	125	6.3
	Back	1,158	58.7
	Side	381	19.3
	Up	53	2.7
	Bottom	211	10.7
	Don't know	44	2.2
4. Do you think packed processed foods are healthy?	Yes	1,090	55.4
	No	684	34.7
	Don't know	195	9.9
5. Do you think packed processed foods are healthy?	Yes	1,090	55.4
	No	684	34.7
	Don't know	195	9.9

More than half of the respondents (51.7%) consumed *namkeen* daily, while less than half (45.7%) consumed cookies or biscuits daily. Maximum proportion of respondents consumed breakfast cereals (34.1%), cake (49.2%), chocolate (46.6%), noodles (42.8%), pasta (49.3%) and packaged juices (38.3%) on a monthly basis (Figure 1). Weekly consumption of aerated beverages was reported by 43.7% of the respondents. Using multivariate analysis of variance (MANOVA), the difference in the consumption frequencies of the items was highly significant (*p*-value = 0.00001). The daily consumption of aerated beverages was more among younger people than older ones and was statistically significant (*p* < 0.05). Significant differences were found in the frequency of consumption of chocolate, noodles, pasta, and aerated beverages with the geographical zone (F-value = 6.955091, *p*-value = 0.00042). All packaged items have significant differences in the frequency of consumption with locality (F-value = 11.068549, *p*-value = 0.000027).

The participant's knowledge of the existing FoP label design practiced worldwide was also assessed in the current study (Table 2). The majority of the respondents (95%) were aware of the food package labeling, and 88.6% of them considered this information helpful. Over half (57.8%) of them reported that the food package labels are present on the back of the food items. A similar proportion (55.4%) of the respondents considered packaged foods as healthy.

**Figure 2:**
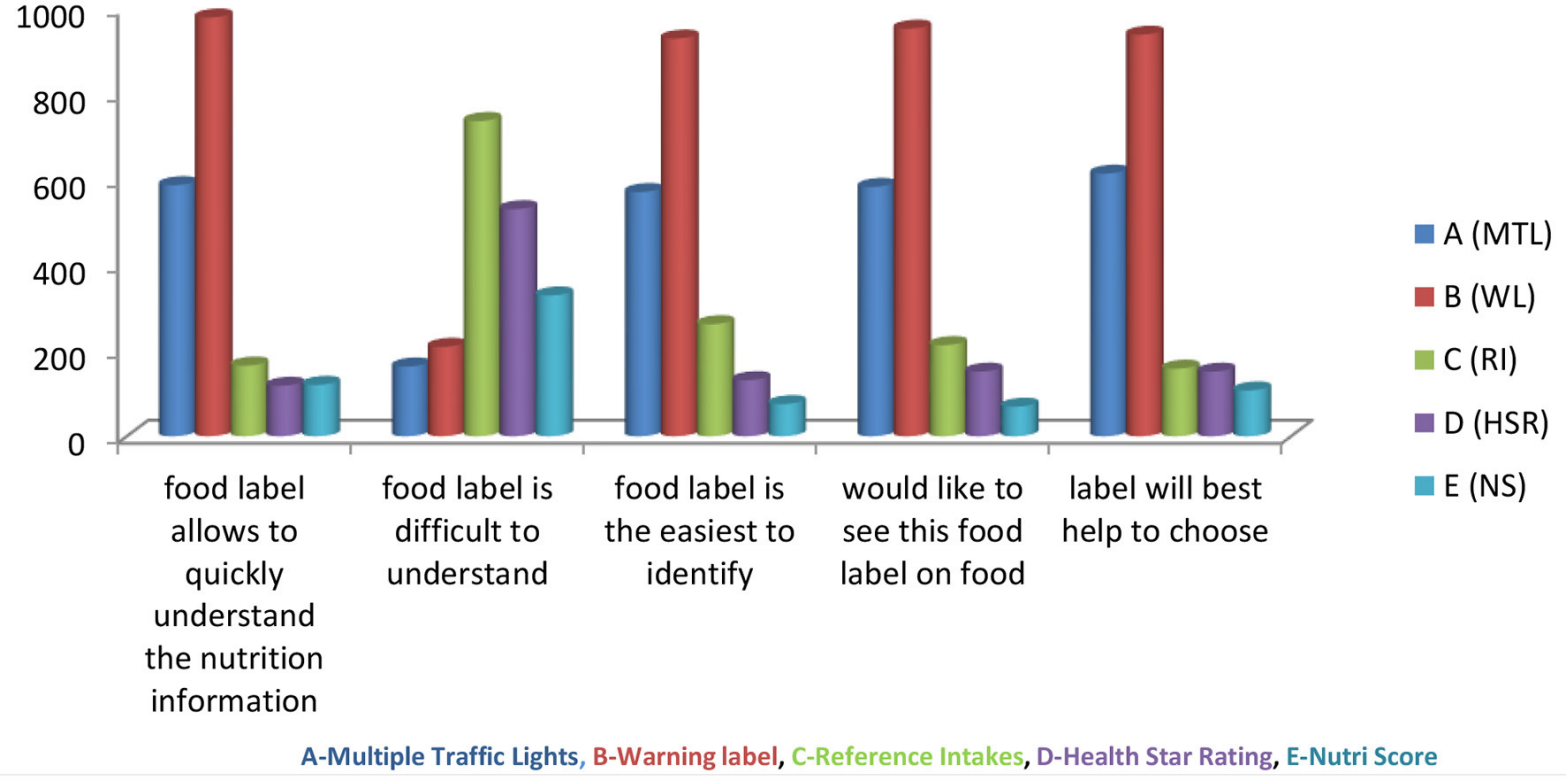
Perceptions about different types of food labeling.

**Figure 3:**
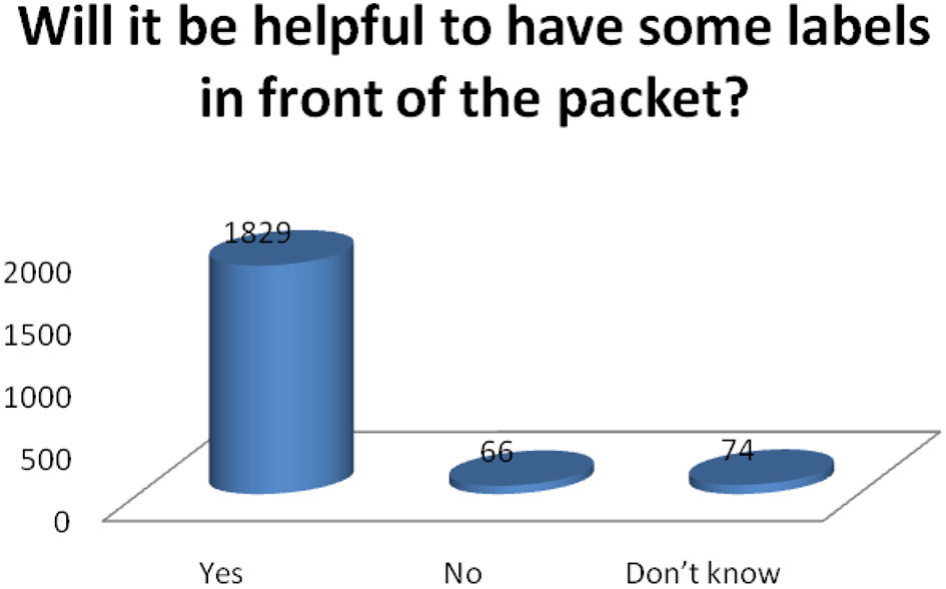
Consumers opinion about front of pack food labels.

Different FoP labeling designs such as Multiple traffic lights (MTL), Warning Labels (WL), Health Star Ratings (HSR), Reference Intake (RI), Nutri Score (NS) were presented to the respondents to determine their perception of these designs. The preferences of the different front of pack labels in terms of comprehensiveness, consumer-friendliness, and identifiability were statistically significant. Warning Labels (WL) were the most preferred food labels (93%), followed by Multiple Traffic Lights (MTL) and the difference between the two was found to be statistically significant (*p*-value < 0.05). All items show a significant difference between MTL and WL (*p*-value < 0.05) (Figure 2). There was a significant difference in Perception of Consumer with respect to gender (HT value = 3.2571, *p*-value = 0.006); education (HT value = 12.969, *p*-value = 0.0001); and employment status (HT value = 2.690, *p*-value = 0.020). The majority of the respondents reported that it would be useful to have the nutrition labels on the front of the packet (Figure 3).

## Discussion

The consumers have low and wrong perceptions about FoP labels in their food items which is not good for the health of the common people. Although India's Food Safety Standards Authority (FSSAI) introduced the nutrition labeling regulations in 2011, further amended in 2018 and 2020, their implementation lacks robustness. Due to the increasing burden of non-communicable diseases and their connection with lifestyle factors, including diet, consumers have become more concerned about what they eat and how much they should eat a particular food product. But the lack of understanding about the effective use of the nutrition labeling is the issue of concern. The current study presents the respondents' awareness, attitude, and behavior about the Front-of-pack labels, recently recommended by Government of India to regulate the intake of junk and processed food (29). The information regarding nutritional content of any packaged food is crucial as they allow the consumer to make the right decision before purchasing it (30).

Reading nutritional content while purchasing and consuming packaged food was found to be more among the younger adults living in North and South India, urban residents, undergraduates, and middle-income groups. Studies suggest that the use of food labels decreases with the increasing age (31, 32). As reported in a study by Andrews et al. (33), older people make less use of nutrition labels as they find them less comprehensible. However, fewer studies depict that nutritional labeling becomes more rigorous with increasing age (34–36). Urbanization influences both quantity and diversity of food consumption in India. The average consumption of packaged and processed foods is higher in urban than rural areas (37).

The majority of the respondents were aware of the food package labeling and considered this information helpful. This finding underlines the significance of nutrition labeling in determining the quality of food products and thus selecting them for better health results (38). Further, nutrition labeling helps make healthier choices by limiting total energy intake (39), fat (40) or sugar (41), thereby reducing the risk of metabolic disorders, etc. (42). The study reports that a higher level of education was significantly associated with a higher level of awareness regarding food labels. Various studies have confirmed the association between higher education and a healthier diet (43, 44). Highly educated consumers find it easier to read and understand the food labels than those having lower education levels (45). We also found that employed people consumed maximum packaged food and beverages as compared to the retired people again this may be due to the busy life of the employed persons.

The daily consumption of aerated beverages was more among younger people than older ones. A study by Yang et al. found more consumption of carbonated soft drinks among young people in the developing nations (46). It was highlighted that consumers look for the information about the available nutrients in the food product before making a decision to purchase it.

Our results suggested that most respondents preferred warning labels on front packs of packaged foods and beverages, indicating excessive salt, sugar or fat levels in the current study. Similar studies show that consumers are receptive to front-of-pack calorie-related information and those with specific risk prefer more specific front-of-pack information on salt, sugar, and fat (47). The study further suggests that the majority of the respondents preferred nutrition labeling on the front of the pack. A study conducted in Europe found that nutritional information given on the front would allow the consumers to compare the food products and make quick decisions regarding its purchase (48). A maximum proportion of the consumption of packaged food was found among the participants from South and North India and it was found to be statistically significant (*p* < 0.005) in terms of the geographical zone. It may be their rapid changing of lifestyle after the COVID restrictions in India. Front-of-pack nutrition labeling is generally considered to be an influential and candid instrument for discouraging the consumption of processed foods, which the Indian policymakers could use to restrain the increasing consumption of processed foods high in fat, sodium, and sugar. Which is responsible for the increasing burden of non-communicable diseases. Various countries like Denmark, Chile, Norway, Singapore, South Africa, Ecuador, etc. are using exciting designs for FoP labeling. The FoP label system lacks uniformity across the globe. Instead, different countries use different labeling systems depending upon their respective feasibility and prevalent socio-demographic profiles of population such as education, general awareness, health and nutrition literacy, and many more (49–53).

## Strengths and Limitations of the Study

The present study was conducted in 14 states of India in all four regions of the country, making the sample representative at the state and national level, hence, providing true estimates of the people's knowledge and attitude on FoP labels across the country. The study has a few limitations. The cross-sectional data doesn't allow exploration of causal pathways behind the observed associations. The authors could not investigate the role of nutrition labeling on purchasing the food products. Furthermore, the survey sampled has substantially fewer women than men, which might affect its gender-specific generalizability.

## Conclusion

The study highlighted that consumers are aware of the nutrition labeling and consider it important while purchasing food products. They also supported the placement of nutrition labeling on the front of the pack. Although Efforts are on to implement the FoP Labeling on the packaged or processed foods at the level of FSSAI, GOI. Taking examples from other countries, more evidence-based research is recommended regarding the knowledge and perception of people on feasibility of FoP label design which may lay a foundation to formulate laws and policies regarding the front of pack labeling.

## Way Forward

A mixed method study in a simulated context in India, we feel, will yield higher fidelity in the future.

## Data Availability Statement

The raw data supporting the conclusions of this article will be made available by the authors, without undue reservation.

## Ethics Statement

The studies involving human participants were reviewed and approved by IEC. The patients/participants provided their written informed consent to participate in this study.

## Author Contributions

All authors listed have made a substantial, direct, and intellectual contribution to the work and approved it for publication.

## Conflict of Interest

OB and VS were employed by Global Health Advocacy Incubator. The remaining author declares that the research was conducted in the absence of any commercial or financial relationships that could be construed as a potential conflict of interest.

## Publisher's Note

All claims expressed in this article are solely those of the authors and do not necessarily represent those of their affiliated organizations, or those of the publisher, the editors and the reviewers. Any product that may be evaluated in this article, or claim that may be made by its manufacturer, is not guaranteed or endorsed by the publisher.
